# Deep Oxidative Desulfurization of Fuels in the Presence of Brönsted Acidic Polyoxometalate-Based Ionic Liquids

**DOI:** 10.3390/molecules25030536

**Published:** 2020-01-26

**Authors:** Argam Akopyan, Ekaterina Eseva, Polina Polikarpova, Anastasia Kedalo, Anna Vutolkina, Aleksandr Glotov

**Affiliations:** 1 Department of Petroleum Chemistry and Organic Catalysis, Moscow State University, 119991 Moscow, Russia; arvchem@yandex.ru (A.A.); esevakatya@mail.ru (E.E.); polikarpova-polina@rambler.ru (P.P.); nastya.kedalo@mail.ru (A.K.); annavutolkina@mail.ru (A.V.); 2 Department of Physical and Colloid Chemistry, Gubkin Russian State University of Oil and Gas, 119991 Moscow, Russia

**Keywords:** oxidative desulfurization, Keggin-type polyoxometalate, ionic liquids, Brönsted acidic

## Abstract

Polyoxometalate-based ionic liquid hybrid materials with a pyridinium cation, containing Brönsted acid sites, were synthesized and used as catalysts for the oxidation of model and real diesel fuels. Keggin-type polyoxometalates with the formulae [PMo_12_O_40_]^3−^, [PVMo_11_O_40_]^4−^, [PV_2_Mo_10_O_40_]^4−^, [PW_12_O_40_]^3−^ were used as anions. It was shown that increasing the acid site strength leads to an increase of dibenzothiophene conversion to the corresponding sulfone. The best results were obtained in the presence of a catalyst, containing a nicotinic acid derivative as cation and phosphomolybdate as anion. The main factors affecting the process consisting of catalyst dosage, temperature, reaction time, oxidant dosage were investigated in detail. Under optimal conditions full oxidation of dibenzothiophene and more than a 90% desulfurization degree of real diesel fuel (initial sulfur content of 2050 ppm) were obtained (the oxidation conditions: NK-1 catalyst, molar ratio H_2_O_2_:S 10:1, molar ratio S:Mo 8:1, 1 mL MeCN, 70 °C, 1 h). The synthesized catalysts could be used five times with a slight decrease in activity.

## 1. Introduction

In recent years, the produced hydrocarbon feedstock has contained an increasing number of organosulfur compounds, which adversely affect the refining processes and the quality of the resulting fuels [1]. Sulfur oxides generated during the combustion of fuels have a negative impact both on the environment and human health. In this regard, in many countries stringent environmental requirements, limiting the sulfur content in light fuels to 10 ppm, have been introduced [2]. Since the increase in sulfur content of oils leads to an increase in the cost of the hydrodesulfurization, the interest of researchers has been increasingly attracted by non-hydrogen desulfurization methods [3,4,5]. Oxidative desulfurization is one of the most promising ones among the non-hydrogen desulfurization methods (extraction, adsorption, biodesulfurization) [1,6,7]. The method of oxidative desulfurization is based on the oxidation of sulfur-containing moieties and subsequent removal thereof from petroleum products [8,9].

The main advantages of oxidative desulfurization are lower temperatures and pressures, as well as the absence of hydrogen [10,11]. Another advantage of oxidative desulfurization is the ability to remove organosulfur compounds not destroyed in the process of hydrodesulfurization [8]. Hydrogen peroxide is the most common oxidant, because only water is formed as a byproduct [12].

Transition metal salts, in particular containing molybdenum, copper, vanadium, and tungsten, have become the most common catalysts for oxidation [13,14,15,16,17]. Such catalysts have become widespread due to their ability to form peroxocomplexes in the presence of hydrogen peroxide [7,18,19]. Interest in oxometallic catalysts is also growing [15]. An important advantage of such catalysts is that they can be used for a minimum of five cycles [16]. The main problem associated with the use of catalysts based on transition metal salts is the presence of two reaction phases: the organic phase with organosulfur compounds, and the polar phase, where oxidant is present. To eliminate phase transfer constraints, interfacial carriers are used, which contribute to a faster and more efficient reaction [20]. Ionic liquids have become widespread as interfacial carriers in the oxidation of sulfur compounds [1,21,22,23,24,25]. For example, the authors of [24] managed to increase the conversion of dibenzothiophene (DBT) from 10% to 100% in 1 h by adding an ionic liquid (IL) to the catalytic mixture. In another article [26], it was shown that more than 90% of DBT can be removed by adding to hydrogen peroxide [(C_2_H_4_OH)N(CH_3_)_3_]FeCl_4_ (ChFeCl_4_) and an ionic liquid (1-butyl-3-methylimidazole tetrafluoroborate [BMIM]BF_4_ or 1-n-octyl-3-methylimidazole tetrafluoroborate [OMIM]BF_4_) as a catalyst. This catalytic system containing the IL and the catalyst can be easily separated and used up to five times without loss of activity. Addition of the ionic liquid Bmim^+^BF_4_^−^ to the commercial catalyst H_3_PW_12_O_40_·14H_2_O allows complete conversion of DBT and 4,6- dimethyldibenzothiophene in 3 h of reaction at 70 °C [27].

As far as is known from the literature, the oxidation of sulfur compounds proceeds easily in acidic media [28,29,30]. Brønsted acids (sulfuric acid, phosphoric acid, formic acid, acetic acid, etc.) are often used as catalysts for oxidation of sulfur compounds [28,31]. Catalytic systems based on ionic liquids containing Brønsted acidity are also known [32,33]. The disadvantage of such catalytic systems is their lower activity compared to catalysts based on molybdenum and tungsten [1,34,35]. In comparison with the above mentioned catalysts. the synthesized system has the following advantages: content of ionic liquid in the catalytic system (0.5 mL of ionic liquid in ref. [24], less than 100 mg in our case) and reaction time (3 h in ref. [27], 1 h in this paper).

The ionic liquids used in this work as catalysts consist of cation-containing Brønsted acidity and an anion containing a transition metal. The Brønsted acid sites are presented by carboxylic groups, bonded with a pyridinium cation and the transition metal containing anion is presented by the phosphoromolybdate anion. The advantage of such an approach is the possibility to provide a single catalyst having the high catalytic activity of transition metals and acids as well as the functions of an interfacial carrier.

## 2. Results and Discussion

### 2.1. Characterization of the Catalysts

The elemental CHN composition of ILs with Bronsted acidity was determined. The results obtained show similar values of experimentally detected and calculated values (weight percentage) which proves the composition of the synthesized catalysts and the proposed molar ratios of corresponding cation and anion. The measurement error did not exceed 1%.

Calc. for Py-1: C, 20.84; H, 1.99; N, 3.47. Found: C, 20.87; H, 1.98; N, 3.53.
Calc. for Py-2: C, 23.02; H, 2.4; N, 3.36. Found: C, 22.95; H, 2.35; N, 3.38.
Calc. for Py-3: C, 28.76; H, 3.49; N, 3.05. Found: C, 27.96; H, 3.27; N, 3.0.
Calc. for NK-1: C, 26.97; H, 3.15; N, 3.15. Found: C, 26.69; H, 3.09; N, 3.17.
Calc. for NK-2: C, 21.78; H, 2.61; N, 2.54. Found: C, 20.97; H, 2.13; N, 2.27.
Calc. for NK-3: C, 10.84; H, 1.49; N, 1.26. Found: C, 10.97; H, 1.63; N, 1.57.
Calc. for NK-4: C, 38.78; H, 5.39; N, 2.51. Found: C, 39.94; H, 5.99; N, 2.37.

^1^H, ^13^C NMR, and ESI-MS spectra are shown as follows (Supporting Materials Figures S1–S15):

*1-(carboxymethyl) pyridinium bromide (*3*):* Yield 80%. ^1^H NMR spectrum (DMSO, 400 MHz): δ = 5.72 (s, 2H), 8.20–8.24 (t, 2H), 8.66–8.7 (t, 1H), 9.18–9.2 (d, 2H). ^13^C NMR δ = 167.6, 146.4, 146.2, 127.7, 60.6 ppm. ESI-MS: positive ion *m*/*z* = 138 [C_5_H_5_NCH_2_COOH]^+^. pK_a_ = 1.9.

*1-(2-carboxyethyl) pyridinium bromide (*4*):* Yield 88%. ^1^H NMR spectrum (DMSO, 400 MHz): δ = 3.07–3.11 (t, 2H), 4.78–4.82 (t, 2H), 8.13–8.15 (t, 2H), 8.57–8.60 (t, 1H), 9.13–9.15 (d, 2H). ^13^C NMR δ = 171.5, 145.8, 145.4, 127.8, 127.1, 56.5, 34.5 ppm. ESI-MS: positive ion *m*/*z* = 152 [C_5_H_5_N(CH_2_)_2_COOH]^+^. pK_a_ = 4.2.

*1-(5-carboxypentyl) pyridinium bromide (*5*):* Yield 86%. ^1^H NMR spectrum (DMSO, 400 MHz): δ = 1.27 (m, 2H), 1.5–1.54 (qt, 2H), 1.91 (qt, 2H), 2.19–2.22 (t, 2H), 4.57–4.61 (t, 2H), 8.14–8.17 (t, 2H), 8.58–8.6 (t, 1H), 9.08–9.10 (d, 2H).^13^C NMR δ = 179.1, 146.2, 144.9, 128.9, 62.5, 34.3, 31.1, 25.5, 24.4 ppm. ESI-MS: positive ion *m*/*z* = 194 [C_5_H_5_N(CH_2_)_5_COOH]^+^. pK_a_ = 4.4.

*1-butyl-3-carboxypyridinium bromide (*6*):* Yield 68%. ^1^H NMR spectrum (DMSO, 400 MHz): δ = 0.88–0.92 (t, 3H), 1.3 (m, 2H), 1.89 (m, 2H), 4.67–4.71 (t, 2H), 8.24–8.27 (t, 1H), 8.93–8.95 (d, 1H), 9.26–9.28 (d, 1H), 9.58 (s, 1H). ^13^C NMR δ = 165.1, 149.3, 148.4, 146.7, 128.2, 126.7, 63.6, 30.3, 19.5, 12.9 ppm. ESI-MS: positive ion *m*/*z* = 180 [C_5_H_4_N(C_4_H_9_)COOH]^+^. pK_a_ = 0.2.

*1-dodecyl-3-carboxypyridinium bromide (*7*):* Yield 48%. ^1^H NMR spectrum (DMSO, 400 MHz): δ = 0.82–0.84 (t, 3H), 1.22 (m, 14H), 1.39 (m, 2H), 1.73 (qt, 2H), 1.9 (m, 2H), 4.36–4.39 (t, 2H), 4.67–4.70 (t, 2H), 8.27 (t, 1H), 8.96–8.98 (d, 1H), 9.28 (d, 1H), 9.59 (s, 1H) ^13^C NMR δ = 164.8, 149.3, 148.3, 146.5, 128.1, 126.6, 64.1, 31.98, 31.6, 30.4, 30.23, 29.58, 29.54, 29.36, 29.27, 28.03, 22.72, 14.10 ppm. ESI-MS: positive ion *m*/*z* = 292 [C_5_H_4_N(C_12_H_25_)COOH]^+^.

Elemental analysis of the metal content in the obtained catalysts with Bronsted acidity was carried out. The results are presented in Table 1. The metal amounts found agree with the theoretically calculated values.

### 2.2. Spectral Characterizations

**Figure 1:**
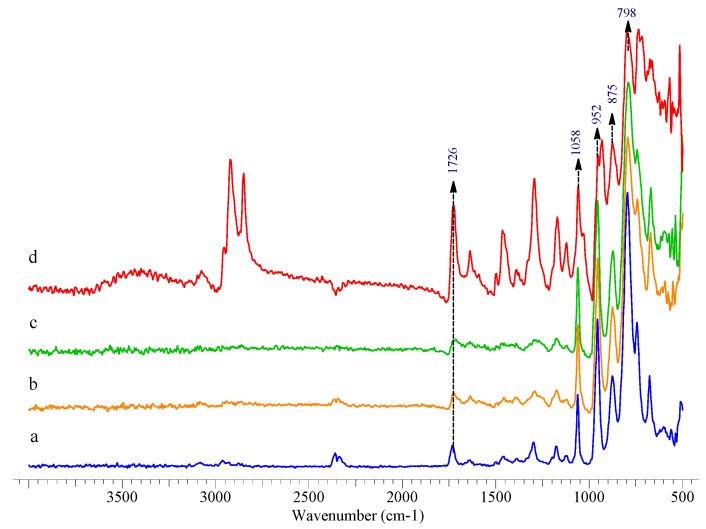
IR spectra of catalysts: (**a**) NK-1, (**b**) NK-2, (**c**) NK-3, (**d**) NK-4.

**Figure 2:**
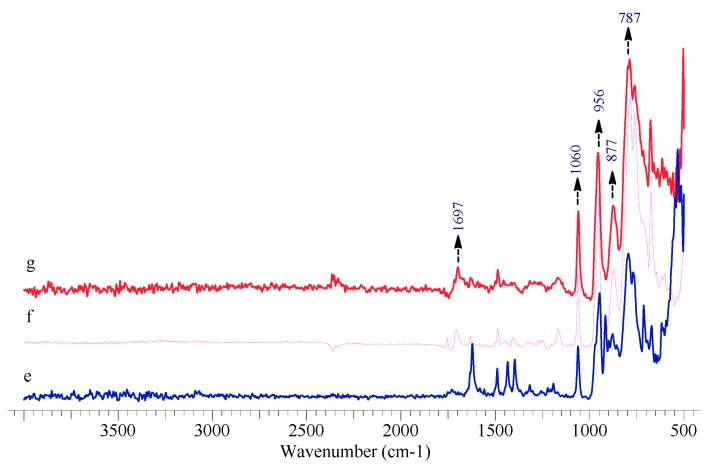
IR spectra of catalysts: (**e**) Py-1, (**f**) Py-2, (**g**) Py-3.

The intermolecular electronic interaction between heteropolyacid anions and IL-cations are illustrated by IR spectra in Figure 1 and Figure 2. The anion [PMo_12_O_42_]^7–^ shows four characteristic peaks at 1058, 952, 875, and 798 cm^−1^, corresponding to the P–O, M = O_a_, M–O_b_–Mo, and Mo–O_c_–Mo bonds (where O_a_ is the terminal oxygen, O_b_—bridging oxygen between corner-sharing octahedral, O_c_—bridging oxygen between edge-sharing octahedral) for the catalysts based on nicotinic acid (Figure 1) and characteristic peaks at 1060, 956, 877, and 787 cm^−1^ for the catalysts based on pyridine (Figure 2). The presence of four characteristic absorption peaks above indicates that the obtained heteropolyacid’ anion based on ILs keeps the Keggin-type structure [36]. There are characteristic absorption peaks of the carboxyl group of these ILs at 1725 and 1697 cm^−1^ respectively. Bands at 2800–3000 cm^−1^ are the stretching vibrations of the saturated C–H bond in NK-4 (Figure 1d). These results indicate the formation of the catalyst containing IL, cation, and heteropolyacid anion.

### 2.3. Catalytic Tests

**Figure 3:**
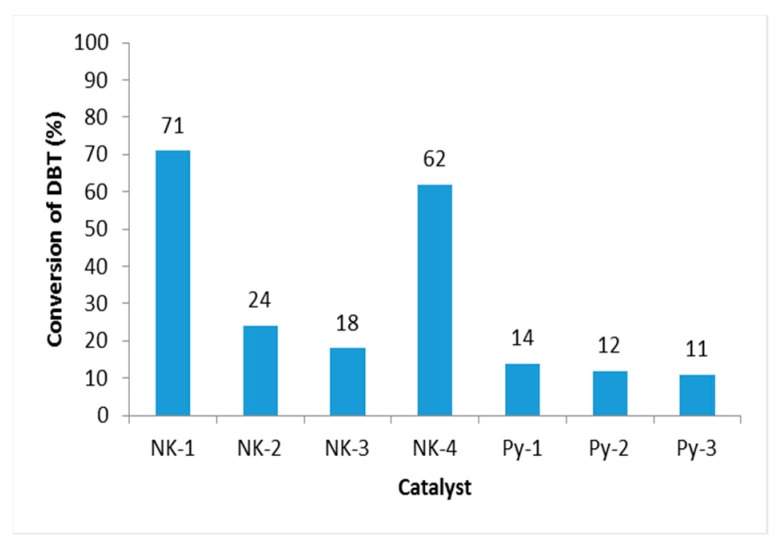
Conversion of dibenzothiophene over various catalysts. Reaction conditions: initial sulfur content 500 ppm, 5 mL of model mixture, molar ratio H_2_O_2_:S = 4:1, molar ratio sulfur: molybdenum 8:1, 1 mL MeCN, 50 °C, 1 h. Conversion of dibenzothiophene without catalyst is 7%.

In order to evaluate the activity of catalysts, the oxidation of model mixtures containing dibenzothiophene was studied under the same conditions (Figure 3).

Among the phosphoromolybdenum-containing ionic liquids, NK-1 and NK-4 appear to be more active catalysts (Figure 3). The results obtained show that the conversion of dibenzothiophene increases with the acidity of the cation (NK-1 > Py-1 > Py-2 > Py-3). Increasing the extent of proton substitution also leads to higher DBT conversion (NK-1 > NK-2 > NK-3). This fact can be connected to the catalytic activity of the carboxylic group by formation of the corresponding peracids due to the multifunctional nature of the synthesized catalysts. The multifunctional nature of the catalyst is explained by the fact that the catalyst used combines several functions such as:

- the formation of peroxocomplexes by anions, which are intended for the oxidation of organosulfur compounds;
- the formation of the corresponding peracid, also capable of oxidizing organosulfur compounds;
- Bronsted acid site for activation of sulfur atoms;
- a cation playing the role of an interphase carrier due to the ability to control hydrophobic properties by varying the length of the alkyl chain.

**Figure 4:**
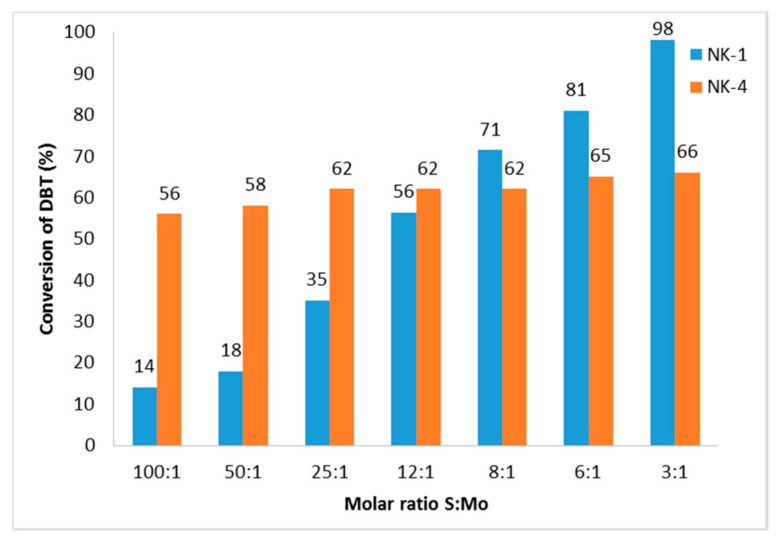
Effect of amounts NK-1 and NK-4 on conversion of dibenzothiophene (DBT). Reaction conditions: initial sulfur content 500 ppm, 5 mL of model mixtures, molar ratio H_2_O_2_:S = 4:1, 1 mL MeCN, 50 °C, 1 h.

Studies of the effect of the catalyst amount show, that a higher content of NK-1 leads to an increase of DBT conversion achieving 98% at molar ratio S/Mo = 3:1 (Figure 4). The results obtained for NK-4 show that the catalyst works even at a very small molar ratio S/Mo = 100:1. This fact may be explained by the good surface activity of NK-4, that is why all the added amount of the catalyst is concentrated on the interface. The increase in the NK-4 amount by 20 times did not lead to significant rise in DBT conversion, which may be explained by a higher decomposition of hydrogen peroxide.

**Figure 5:**
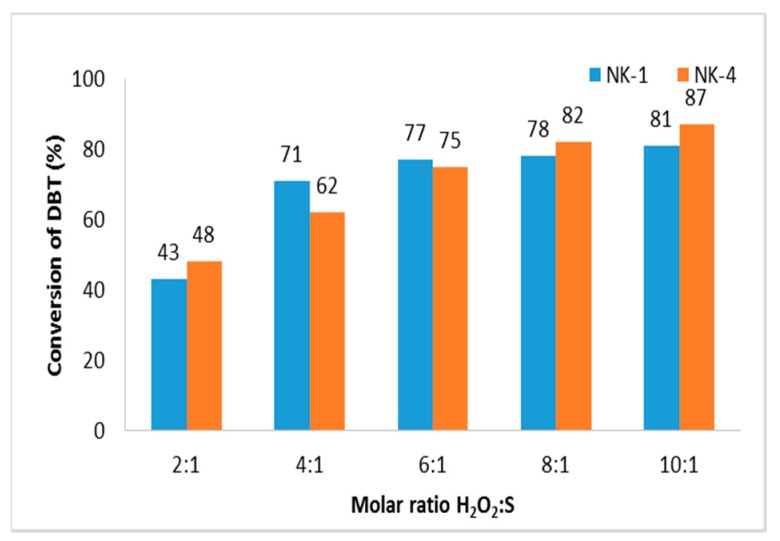
Effect of the amount of hydrogen peroxide on conversion of the DBT. Reaction condition: total sulfur 500 ppm, 5 mL of model mixtures, molar ratio S:Mo 8:1, 1 mL MeCN, 50 °C, 1 h.

Effect of hydrogen peroxide amount was studied for catalysts NK-1 and NK-4 at temperature 50 °C (Figure 5).

For NK-1 catalyst an increase in hydrogen peroxide amount from molar ratio H_2_O_2_:S 4:1 to 10:1 did not lead to a significant rise in conversion of DBT which indicates the limitations connected with phase transfer restrictions, but for catalyst NK-4 having less transfer limitations, an increase of hydrogen peroxide dosage allowed better results to be obtained, which also indicates the possible decomposition of oxidant in the presence of NK-4.

**Figure 6:**
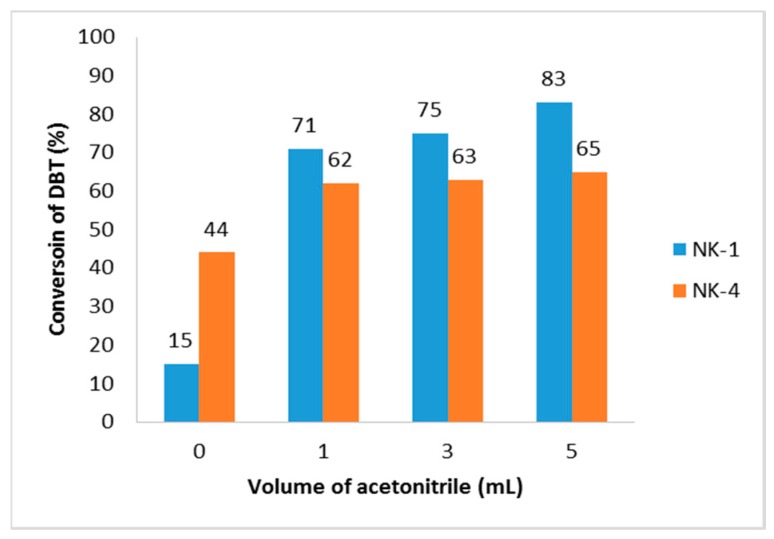
Effect of the acetonitrile amount on the conversion of the DBT. Reaction conditions: initial sulfur content 500 ppm, 5 mL of model mixture, molar ratio H_2_O_2_:S 4:1, molar ratio S:Mo 8:1, 50 °C, 1 h.

Variation of the amount of acetonitrile shows that NK-1 is sensitive to the presence of acetonitrile which plays the role of extractant and solvent both for catalyst and oxidant. It should be noted that NK-4, having better lipophilic properties, is less sensitive to the presence of solvent (Figure 6).

**Figure 7:**
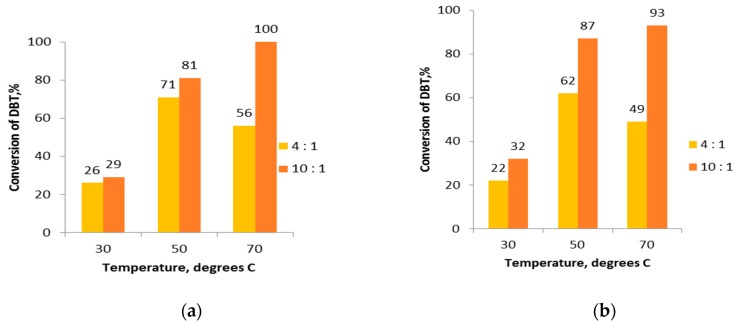
Effect of temperature on the conversion of the DBT in the presence of NK-1 catalyst (**a**) and NK-4 catalyst (**b**). Reaction conditions: initial sulfur content 500 ppm, 5 mL of model mixture, molar ratio S:Mo 8:1, molar ratio H_2_O_2_:S 4:1, 1 mL MeCN, 1 h.

The temperature dependence of DBT conversion was studied for both NK-1 and NK-4 catalysts under two types of hydrogen peroxide dosage (molar ratio oxidant:sulfur 4:1 and 10:1) to understand the thermal decomposition of hydrogen peroxide (Figure 7).

**Figure 8:**
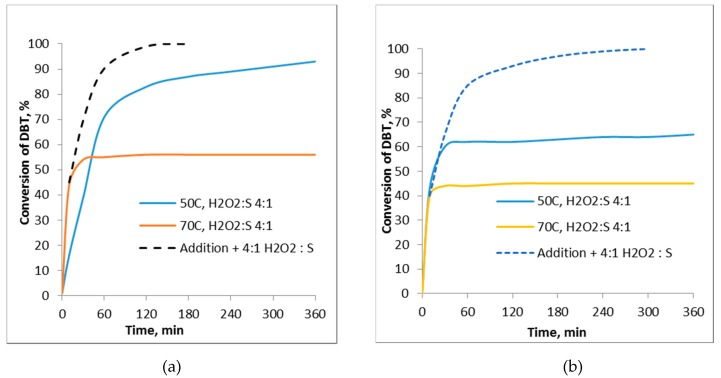
Effect of the oxidation duration on the conversion of the DBT in the presence of NK-1 catalyst (**a**) and NK-4 catalyst (**b**). Reaction conditions: initial sulfur content 500 ppm, 5 mL of model mixture, NK-1 as catalyst, molar ratio H_2_O_2_:S 4:1 (10:1), molar ratio S:Mo 8:1, 1 mL MeCN.

The results obtained show that complete oxidation of DBT can be achieved by using NK-1 catalyst at 70 °C and oxidant:sulfur 10:1 molar ratio. While using four times excess of oxidant for NK-1 and NK-4, increasing the temperature from 50 to 70 °C leads to a decrease in DBT conversion which probably indicates the decomposition of hydrogen peroxide at higher temperature. At the same time, increasing the oxidant amount allows a high conversion of the substrate to be obtained at 70 °C (100% for NK-1 catalyst and 93% for NK-4). The time dependence of DBT conversion obtained is shown in Figure 8.

The results obtained confirm that the catalyst plays a significant role in the hydrogen peroxide decomposition at temperature 70 °C. Thus, for both catalysts the corresponding kinetic curves reach a plateau after 10 min of the reaction and additional dosage of oxidant allows continuation of the oxidation reaction to achieve more than 90% conversion of DBT. It should be noted that decomposition of hydrogen peroxide has higher rates for the NK-4 catalyst, which does not allow achievement of 100% conversion even after addition of an extra amount of oxidant. Also, for catalyst NK-4 the kinetic curve reaches a plateau even at temperature 50 °C while NK-1 works after 6 h of the reaction at the same temperature. Thus, despite the catalyst NK-4 reaching the plateau faster than NK-1 due to better hydrophobic properties, for complete oxidation of DBT it is preferable to use NK-1.

The results of the study of oxidation for different classes of organosulfur compounds are shown in Table 2.

The results obtained show that the oxidation activity of substrate decreases in the order sulfides > 4-methyldibenzothiophene > dibenzothiophene > 4,6-dimethyldibenzothiophene > benzothiophene > 5-methylbenzothiophene which correlates with literature data [10,37].

**Figure 9:**
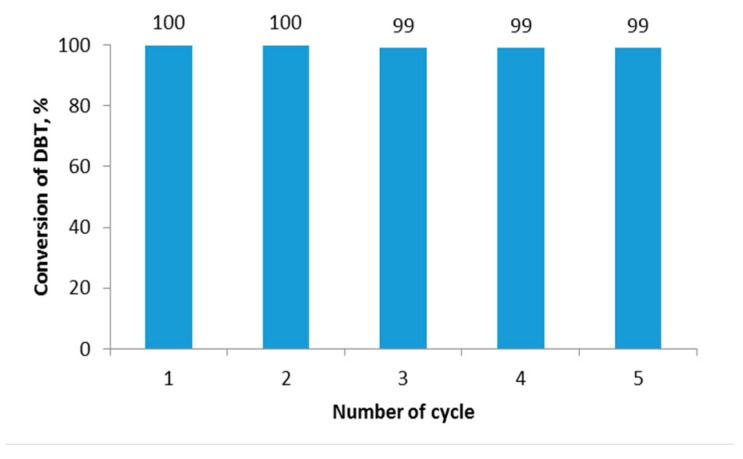
Effect of recycling NK-1 on the conversion of DBT. Reaction conditions: initial sulfur content 500 ppm, 5 mL of model mixture, molar ratio H_2_O_2_:S 10:1, molar ratio S:Mo 8:1, 1 mL MeCN, 70 °C, 1 h.

The possibility of catalyst reuse by recycling was studied for NK-1 (Figure 9).

Upon completion of reaction the catalyst was separated by centrifuging and used in the oxidation of a new portion of model mixture. The results obtained shows that the catalyst maintains its activity even after 5 cycles.

Oxidation of model sulfides shows that the NK-1 catalyst is most effective in this reaction. Activity of this catalyst was investigated in oxidative desulfurization of real diesel fraction (initial sulfur content of 2050 ppm). After oxidation, the reaction mixture was passed through silica gel to remove oxidized sulfur compounds. The oxidation of the diesel fraction was carried out under the most effective conditions selected for the oxidation of model sulfide mixtures: molar ratio H_2_O_2_:S, 10:1, molar ratio S:Mo 8:1, 1 mL MeCN, 70 °C, 1 h. As a result of oxidation, the degree of desulfurization of the diesel fraction was 92%. The degree of desulfurization during oxidation without a catalyst was 15%. Based on the obtained data, it can be concluded that the synthesized catalysts are effective catalysts for oxidative desulfurization.

## 3. Experimental

### 3.1. Materials

The ionic liquids and the model mixtures were synthesized from ethyl bromoacetate (99%, Sigma Aldrich, Steinheim, Germany), bromopropionic acid (97%, Sigma Aldrich, Steinheim, Germany), bromhexanoic acid (97%, Sigma Aldrich, Steinheim, Germany), bromobutane (99%, Sigma Aldrich, Steinheim, Germany), 1-bromododecane (98%, Acros Organics, Steinheim, Germany), pyridine (99,95%, Komponent-Reaktiv, Moscow, Russia), nicotinic acid (99%, Chemical Line, St.Petersburg, Russia), hydrochloric acid (37%, Sigma-Tek, Moscow, Russia), acetonitrile (99,85%, Komponent-Reaktiv, Moscow, Russia), dibenzothiophene (DBT, 98%, Sigma Aldrich, Steinheim, Germany), benzothiophene (98%, Sigma Aldrich, Steinheim, Germany), methyl phenyl sulfide (99%, Acros Organics, Geel, Belgium), dibenzyl sulfide (95%, Sigma Aldrich, Steinheim, Germany), 5- methylbenzothiophene (97%, Sigma Aldrich, Steinheim, Germany), 4-methyldibenzothiophene (96%, Sigma Aldrich, Steinheim, Germany), 4,6-dimethyldibenzothiophene (95%, Sigma Aldrich, Steinheim, Germany), dodecan (99%, Sigma Aldrich, Steinheim, Germany), ethanol (96%, Moscow, Russia), sodium phosphoromolybdate (Na_7_PMo_12_O_42_, 98%, Sigma Aldrich, Steinheim, Germany), phosphoromolybdic acid (H_7_PMo_12_O_42_*xH_2_0, 99%, Sigma Aldrich, Steinheim, Germany), sodium hydroxide (KhimMed, Moscow, Russia), hydrogen peroxide (H_2_O_2_, 50%, Prime Chemicals Group, Moscow, Russia).

### 3.2. Synthesis of Ionic Liquids

**Scheme 1:**
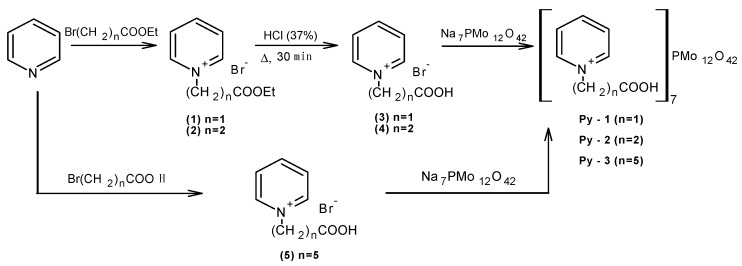
The synthesis of ionic liquids (ILs) based on pyridine.

**Scheme 2:**
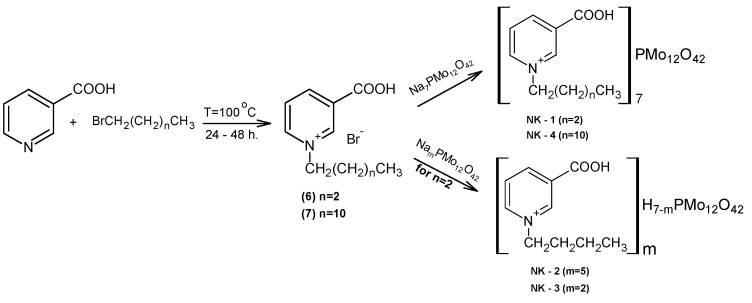
Synthesis of ILs based on nicotinic acid.

Synthesis of ionic liquids (ILs) having the cation-containing Brønsted acidity and the heteropolyacid’s anion used as catalysts for oxidation of sulfur compounds are presented in Scheme 1 and Scheme 2.

Stage 1. The quaternization reactions of the ILs *(1)–(2)* were performed according to the literature procedure [38]. Equimolar amounts of pyridine, 1 mol and bromocarboxylic ethyl ester, 1 mol (or the corresponding ester) were placed in a flask equipped with a water-cooled condenser and magnetic stirrer. The mixture was stirred for 3–6 h at room temperature. After that, the mixture was heated in an oil bath to 80 °C. The product was isolated, washed with benzene (3 × 30 mL). Then, the resulting product was dissolved in methanol, followed by extraction with petroleum ether (3 × 30 mL) and vacuum drying at 30 °C for 24 h. Synthesis of product *(5)* was similar to the procedure [39]. The reaction mixture was refluxed in a flask for 9 h, then cooled down to 20 °C and washed similarly to *(1)–(2)*.

Stage 2. Hydrolysis of products *(1)–(2)* was carried out by refluxing for 30 min using a double excess of the concentrated hydrochloric acid. The solvent was removed in vacuo. The resulting solid *(3)–(4)* was washed with acetone (3 × 30 mL), diethyl ether (3 × 30 mL), and dried in vacuo for 24 h to constant weight.

Stage 3. The catalysts Py-1, Py-2, Py-3 were prepared according to the procedure described previously [40]. The reaction of the anion exchange with sodium phosphoromolybdate was performed using molar ratio 7:1 of IL *(3)* and Na_7_PMo_12_O_42_. A solution of IL (7 mmol) in deionized water was added dropwise to an aqueous solution of sodium phosphoromolybdate (1 mmol) under vigorous stirring at room temperature. The precipitate formed was separated, washed with deionized water and ethyl alcohol, and dried in vacuo for 24 h.

Stage 1. The quaternization of IL *(6)–(7)* was carried out similarly to the methods described in the literature [41,42]. Nicotinic acid (1 mol) and acetonitrile (50 mol) were placed in an autoclave (sealed tube). Then, alkylbromide (3 mol) was added to the resulting suspension. To obtain products *(6)* and *(7)*, the reaction mixture was stirred at 100 °C for 24 h and 48 h respectively. After cooling down to 0 °C for removal of unreacted nicotinic acid, the solution was decanted and dried at 40 °C in vacuo to constant weight. The resulting precipitate *(6)* was dissolved in deionized water, washed with hexane (3 × 15 mL) and diethyl ether (3 × 15 mL) and dried in vacuo for 24 h. The resulting solid *(7)* was dissolved in benzene at 60 °C, filtered off from the precipitate and evaporated using a rotary evaporator at 40 °C. Then product *(7)* was washed with petroleum ether (5 × 30 mL) and dried in vacuo at 50 °C for 24 h.

Stage 2. The catalysts NK-1, NK-2, NK-3 and NK-4 were obtained by the reaction of the anion exchange of IL *(6)–(7)* with Na_7_PMo_12_O_42_.

The sodium salt of phosphoromolybdic acid was prepared as follows. A saturated sodium hydroxide solution was added to a solution of phosphoromolybdenum acid using alkali/acid molar ratios of 7:1 for NK-1 and NK-4, 2:1 for NK-3, and 5:1 for NK-2. Separately, the solutions of IL *(6)* in deionized water and *(7)* in ethanol were prepared. For NK-4, sodium phosphoromolybdate was prepared in ethanol. The IL was added dropwise to the sodium salt and stirred at room temperature for 24 h. The precipitate was separated, washed with ethanol and dried in vacuo for 24 h.

### 3.3. Catalyst Characterization

Infrared spectra (IR) of catalysts were recorded on a Nicolet (Thermo Scientific, Waltham, Massachusetts, USA) IR-2000 instrument in the range of 500–4000 cm^−1^ by the method of multiple violation of total internal reflection using a Multi-reflection HATR attachment containing a 45^o^ ZnSe crystal for various wavelength ranges with a resolution of 4 nm. NMR spectra of ^1^H and ^13^C were measured on a Varian-XR-400 spectrometer (Bruker Avance 400, Watertown, MA, USA) with an operating frequency of 400 MHz. Electrospray ionization mass spectrum (ESI-MS) were recorded on a Dionex Ultimate 3000 (Dionex corporation, Sunnyvale, CA, USA and Berlin, Germany) spectrometer liquid chromatography equipped with an AB Sciex 3200 Qtrap (AB Sciex, Redwood, CA, USA) tandem quadrupole mass-spectrometric detector for the electrospray ionization (ESI) in positive ion recording mode. Elemental analysis of metals in catalysts was performed on an ARL PerformX X-ray fluorescence wave spectrometer (Thermo Fisher Scientific, Waltham, Massachusetts, US). The elemental analysis of carbon, hydrogen, and nitrogen was carried out on a CHNS instrument Thermo Flash 2000 (Thermo Fisher Scientific, GB). The calculation of pKa was carried out according to formula (1), by measuring the pH values of the ionic liquids aqueous solutions at certain concentrations using an electronic pH meter. The concentration of an aqueous solution of ionic liquid was determined by acid–base titration. The pKa values were determined only for water soluble ILs.

pH = ½ × (pK_a_ + pC) (1)

### 3.4. Catalytic Test

Solutions of methylphenylsulfide, dibenzylsulfide, benzothiophene, 5-methylbenzothiophene, dibenzothiophene (DBT), 4-methyldibenzothiophene, and 4,6-dimethyldibenzothiophene in dodecane were used as model oils, with an initial sulfur content 500 ppm. Also model mixtures of DBT with a total sulfur content of 1000 ppm, 2000 ppm were prepared. The oxidative desulfurization catalytic tests were conducted in a reactor equipped with a stirrer, thermostatic water bath, and condenser. The catalyst, H_2_O_2,_ and acetonitrile were added into the reactor, and then 5 mL of a model mixture was injected. The resulting mixture was stirred for 10–360 min at 50 to 70 °C, and after reaction, the upper oil layer was decanted at room temperature and analyzed by GC-FID with an internal standard (Kristall 2000 M equipped with a capillary column (ZB-1 liquid phase, 30 m × 0.32 mm). The solution of catalyst in acetonitrile (lower phase) was collected and used in the recycling experiments.

Oxidation of a real diesel fraction (initial sulfur content—2050 ppm) was performed by the following procedure: NK-1 catalyst (molar ratio Mo:S = 8:1), 1 mL of acetonitrile and 29.5 μL of hydrogen peroxide were added to 10 mL of fuel. The reaction was carried out for 1 h at 70 °C. After oxidation, the reaction mixture was passed through silica gel to remove the oxidized products. The total sulfur content in the hydrocarbon fractions was determined using a sulfur analyzer ASE-2 (analyzer of sulfur energy dispersion) according to the ASTM D4294-10 standard (Standard Test Method for Sulfur in Petroleum and Petroleum Products by Energy Dispersive Xray Fluorescence Spectrometry) [43]. The method is based on X-ray fluorescence energy–dispersive spectrometry to determine the sulfur mass fraction in diesel and unleaded gasoline in the range from 7 to 50 000 ppm with a relative error of 3%.

Each experiment was made repeatedly to obtain a minimum of three convergent results, which differ from the average value less than 5%. The average values were reported in Figure 3, Figure 4, Figure 5, Figure 6, Figure 7, Figure 8, Figure 9 and in Table 2. The measurement error is less than 5%.

## 4. Conclusions

In this work, seven polyoxometallate based-ILs catalysts, including those with Bronsted acid sites presented by carboxylic groups, were synthesized and used as catalysts for the oxidation of organosulfur substrates. It was found that the activity was higher for catalysts containing Bronsted acid sites in the cation. At the same time, it was shown that the catalytic activity depended on the strength of the corresponding acid and was highest for NK-1, containing the carboxylic group directly connected with the pyridinium cation. The influence of reaction conditions (reaction time, oxidant and catalyst amount) on the conversion of DBT was investigated. Thus the increase of NK-1 catalyst content leads to a significant increase of DBT conversion and achieves 98% at molar ratio S:Mo 3:1. The optimal molar ratio range of hydrogen peroxide to total sulfur content is H_2_O_2_:S from 4:1 to 10:1 and depends on reaction temperature. The higher reaction temperatures need higher oxidant dosage for effective oxidation due to the increase of the hydrogen peroxide decomposition rate. Also, it was shown that NK-1 catalyst is sensitive to the presence of acetonitrile which plays the role of extractant and solvent for catalyst, oxidant, and the DBT oxidation products. The conditions for complete oxidation of DBT were found as follows: NK-1 catalyst, molar ratio H_2_O_2_:S 10:1, molar ratio S:Mo 8:1, 1 mL MeCN, 70 °C, 1 h. It was shown that the catalyst synthesized can operate for five cycles without any decrease in its activity.

## Figures and Tables

**Table 1:** The content of molybdenum and phosphorus in ionic liquids according to elemental analysis data.

Ionic Liquid	Designation	Calculated Values, % Mass.		Found Amount, % Mass.	
		*Mo*	*P*	*Mo*	*P*
(NKBu)_7_PMo_12_O_42_	NK – 1	36.98	0.99	37.44 ± 0.31	1.29 ± 0.1
(NKBu)_5_H_2_PMo_12_O_42_	NK – 2	41.81	1.12	41.48 ± 0.3	1.45 ± 0.11
(NKBu)_2_H_5_PMo_12_O_42_	NK – 3	52.01	1.40	51.04 ± 0.27	1.54 ± 0.13
(NKC_12_)_7_PMo_12_O_42_	NK – 4	29.55	0.81	27.61 ± 0.3	1.24 ± 0.08
(PyAc)_7_PMo_12_O_42_	Py – 1	40.84	1.10	40.64 ± 0.31	1.27 ± 0.11
(PyPr)_7_PMo_12_O_42_	Py – 2	39.47	1.06	37.57 ± 0.31	1.20 ± 0.09
(PyHex)_7_PMo_12_O_42_	Py – 3	35.85	0.96	37.40 ± 0.31	1.08 ± 0.1

**Table 2:** Oxidation of different classes of organosulfur compounds. Reaction conditions: total sulfur 500 ppm, 5 mL of model mixtures, NK-1 as catalyst, molar ratio H_2_O_2_:S = 4:1, molar ratio S:Mo 8:1, 1 mL MeCN, 1 h.

Substrate	Conversion, %
methylphenylsulfide	100
dibenzylsulfide	100
benzothiophene	51
5-methylbenzothiophene	25
Dibenzothiophene	71
4-methyldibenzothiophene	96
4,6-dimethyldibenzothiophene	64

## Supplementary Materials

Supplementary are available online at https://www.mdpi.com/1420-3049/25/3/536/s1.

## References

[1] Zhao H., Baker G.A. (2015). Oxidative desulfurization of fuels using ionic liquids: A review. Front. Chem. Sci. Eng..

[2] Bhutto A.W., Abro R., Gao S.R., Abbas T., Chen X.C., Yu G.R. (2016). Oxidative desulfurization of fuel oils using ionic liquids: A review. J. Taiwan Inst. Chem. Eng..

[3] Babich I.V., Moulijn J.A. (2003). Science and technology of novel processes for deep desulfurization of oil refinery streams: A review. Fuel.

[4] Houda S., Lancelot C., Blanchard P., Poinel L., Lamonier C. (2018). Oxidative Desulfurization of Heavy Oils with High Sulfur Content: A Review. Catalysts.

[5] Akopyan A.V., Fedorov R.A., Andreev B.V., Tarakanova A.V., Anisimov A.V., Karakhanov E.A. (2018). Oxidative Desulfurization of Hydrocarbon Feedstock. Russ. J. Appl. Chem..

[6] Qiu L., Cheng Y., Yang C.P., Zeng G.M., Long Z.Y., Wei S.N., Zhao K., Luo L. (2016). Oxidative desulfurization of dibenzothiophene using a catalyst of molybdenum supported on modified medicinal stone. Rsc Adv..

[7] Mokhtar W.N.A.W., Abu Bakar W.A.W., Ali R., Kadir A.A.A. (2018). Development of bimetallic and trimetallic oxides doped on molybdenum oxide based material on oxidative desulfurization of diesel. Arab. J. Chem..

[8] Campos-Martin J.M., Capel-Sanchez M.C., Perez-Presas P., Fierro J.L.G. (2010). Oxidative processes of desulfurization of liquid fuels. J. Chem. Technol. Biotechnol..

[9] Jiang Z.X., Lue H.Y., Zhang Y.N., Li C. (2011). Oxidative desulfurization of fuel oils. Chin. J. Catal..

[10] Polikarpova P., Akopyan A., Shigapova A., Glotov A., Anisimov A., Karakhanov E. (2018). Oxidative Desulfurization of Fuels Using Heterogeneous Catalysts Based on MCM-41. Energy Fuels.

[11] Subhan S., Rahman A.U., Yaseen M., Rashid H.U., Ishaq M., Sahibzada M., Tong Z.F. (2019). Ultra-fast and highly efficient catalytic oxidative desulfurization of dibenzothiophene at ambient temperature over low Mn loaded Co-Mo/Al_2_O_3_ and Ni-Mo/Al_2_O_3_ catalysts using NaClO as oxidant. Fuel.

[12] Campos-Martin J.M., Blanco-Brieva G., Fierro J.L.G. (2006). Hydrogen peroxide synthesis: An outlook beyond the anthraquinone process. Angew. Chem. -Int. Ed..

[13] Al-Shahrani F., Xiao T.C., Llewellyn S.A., Barri S., Jiang Z., Shi H.H., Martinie G., Green M.L.H. (2007). Desulfurization of diesel via the H_2_O_2_ oxidation of aromatic sulfides to sulfones using a tungstate catalyst. Appl. Catal. B-Environ..

[14] Martínez-Vargas D.X., De La Rosa J.R., Sandoval-Rangel L., Guzmán-Mar J.L., Garza-Navarro M.A., Lucio-Ortiz C.J., De Haro-Del Río D.A. (2017). 5-Hydroxymethylfurfural catalytic oxidation under mild conditions by Co (II), Fe (III) and Cu (II) Salen complexes supported on SBA-15: Synthesis, characterization and activity. Appl. Catal. Ageneral.

[15] Li J.-K., Xu Y.-Q., Hu C.-W. (2015). In situ synthesis of a novel dioxidovanadium-based nickel complex as catalyst for deep oxidative desulfurization with molecular oxygen. Inorg. Chem. Commun..

[16] Julião D., Gomes A.C., Cunha-Silva L., Pillinger M., Lopes A.D., Valença R., Ribeiro J.C., Gonçalves I.S., Balula S.S. (2019). Dichlorodioxomolybdenum(VI) complexes bearing oxygen-donor ligands as catalysts for oxidative desulfurization of simulated and real diesel. Catal. Commun..

[17] Komintarachat C., Trakarnpruk W. (2006). Oxidative desulfurization using polyoxometalates. Ind. Eng. Chem. Res..

[18] Rakhmanov E.V., Tarakanova A.V., Valieva T., Akopyan A.V., Litvinova V.V., Maksimov A.L., Anisimov A.V., Vakarin S.V., Semerikova O.L., Zaikov Y.P. (2014). Oxidative desulfurization of diesel fraction with hydrogen peroxide in the presence of catalysts based on transition metals. Pet. Chem..

[19] Rafiee E., Rezaei S. (2016). Deep extractive desulfurization and denitrogenation of various model oils by H_3+n_PMo_12-n_V_n_O_40_ supported on silica-encapsulated gamma-Fe_2_O_3_ nanoparticles for industrial effluents applications. J. Taiwan Inst. Chem. Eng..

[20] Jiang X., Li H.M., Zhu W.S., He L.N., Shu H.M., Lu J.D. (2009). Deep desulfurization of fuels catalyzed by surfactant-type decatungstates using H_2_O_2_ as oxidant. Fuel.

[21] Lu H., Ren W., Wang H., Wang Y., Chen W., Suo Z. (2013). Deep desulfurization of diesel by ionic liquid extraction coupled with catalytic oxidation using an Anderson-type catalyst [(C_4_H_9_)_4_N]_4_NiMo_6_O_24_H_6_. Appl. Catal. A-Gen..

[22] Ibrahim M.H., Hayyan M., Hashim M.A., Hayyan A. (2017). The role of ionic liquids in desulfurization of fuels: A review. Renew. Sustain. Energy Rev..

[23] Akopyan A.V., Eseva E.A., Polikarpova P.D., Baigil’diev T.M., Rodin I.A., Anishnov A.V. (2019). Catalytic Activity of Polyfunctional Ionic Liquids in Oxidation of Model Sulfur Organic Compounds. Russ. J. Appl. Chem..

[24] Hao L., Sun L., Su T., Hao D., Liao W., Deng C., Ren W., Zhang Y., Lü H. (2019). Polyoxometalate-based ionic liquid catalyst with unprecedented activity and selectivity for oxidative desulfurization of diesel in [Omim]BF_4_. Chem. Eng. J..

[25] Lu L., Cheng S., Gao J., Gao G., He M. (2007). Deep oxidative desulfurization of fuels catalyzed by ionic liquid in the presence of H_2_O_2_. Energy Fuels.

[26] Jiang W., Zhu W. (2014). Mechanism and optimization for oxidative desulfurization of fuels catalyzed by Fenton-like catalysts in hydrophobic ionic liquid. J. Mol. Catal. A: Chem..

[27] Li H., He L., Lu J., Zhu W., Jiang X., Wang Y., Yan Y. (2009). Deep oxidative desulfurization of fuels catalyzed by phosphotungstic acid in ionic liquids at room temperature. Energy Fuels.

[28] Yazu K., Makino M., Ukegawa K. (2004). Oxidative desulfurization of diesel oil with hydrogen peroxide in the presence of acid catalyst in diesel oil/acetic acid biphasic system. Chem. Lett..

[29] Zhu Y.F., Zhu M.Y., Kang L.H., Yu F., Dai B. (2015). Phosphotungstic acid supported on mesoporous graphitic carbon nitride as catalyst for oxidative desulfurization of fuel. Ind. Eng. Chem. Res..

[30] Pham X.N., Tran D.L., Pham T.D., Nguyen Q.M., Thi V.T.T., Van H.D. (2018). One-step synthesis, characterization and oxidative desulfurization of 12-tungstophosphoric heteropolyanions immobilized on amino functionalized SBA-15. Adv. Powder Technol..

[31] Krivtsov E.B., Golovko A.K. (2014). The kinetics of oxidative desulfurization of diesel fraction with a hydrogen peroxide-formic acid mixture. Pet. Chem..

[32] Liu D., Gui J., Song L., Zhang X., Sun Z. (2008). Deep desulfurization of diesel fuel by extraction with task-specific ionic liquids. Pet. Sci. Technol..

[33] Gui J.Z., Liu D., Sun Z.L., Liu D.S., Min D., Song B., Peng X.L. (2010). Deep oxidative desulfurization with task-specific ionic liquids: An experimental and computational study. J. Mol. Catal. A-Chem..

[34] Zhu W.S., Li H.M., Jiang X., Yan Y.S., Lu J.D., He L.N., Xia J.X. (2008). Commercially available molybdic compound-catalyzed ultra-deep desulfurization of fuels in ionic liquids. Green Chem..

[35] Kulkarni P.S., Afonso C.A.M. (2010). Deep desulfurization of diesel fuel using ionic liquids: Current status and future challenges. Green Chem..

[36] RocchiccioliDeltcheff C., Aouissi A., Bettahar M., Launay S., Fournier M. (1996). Catalysis by 12-molybdophosphates: 1. Catalytic reactivity of 12-molybdophosphoric acid related to its thermal behavior investigated through IR, Raman, polarographic, and X-ray diffraction studies: A comparison with 12-molybdosilicic acid. J. Catal..

[37] Torres-García E., Galano A., Rodriguez-Gattorno G. (2011). Oxidative desulfurization (ODS) of organosulfur compounds catalyzed by peroxo-metallate complexes of WO_x_–ZrO_2_: Thermochemical, structural, and reactivity indexes analyses. J. Catal..

[38] Thorsteinsson T., Masson M., Kristinsson K.G., Hjalmarsdottir M.A., Hilmarsson H., Loftsson T. (2003). Soft antimicrobial agents: Synthesis and activity of labile environmentally friendly long chain quaternary ammonium compounds. J. Med. Chem..

[39] Chevalier A., Zhang Y.M., Khdour O.M., Hecht S.M. (2016). Selective Functionalization of Antimycin A Through an N-Transacylation Reaction. Org. Lett..

[40] Zhu W.S., Huang W.L., Li H.M., Zhang M., Jiang W., Chen G.Y., Han C.R. (2011). Polyoxometalate-based ionic liquids as catalysts for deep desulfurization of fuels. Fuel Process. Technol..

[41] Chang J.C., Yang C.H., Yang H.H., Hsueh M.L., Ho W.Y., Chang J.Y., Sun I.W. (2011). Pyridinium molten salts as co-adsorbents in dye-sensitized solar cells. Sol. Energy.

[42] Ford L., Ylijoki K.E.O., Garcia M.T., Singer R.D., Scammells P.J. (2015). Nitrogen-Containing Ionic Liquids: Biodegradation Studies and Utility in Base-Mediated Reactions. Aust. J. Chem..

[43] ASTM International (2010). ASTM D4294-10, Standard Test Method for Sulfur in Petroleum and Petroleum Products by Energy Dispersive Xray Fluorescence Spectrometry.

