# Supplementary material for: Deep Oxidative Desulfurization of Fuels in the Presence of Brönsted Acidic Polyoxometalate-Based Ionic Liquids
Source: Molecules. 2020 Jan 26;25(3):536. doi: 10.3390/molecules25030536 (PMC7037028; doi:10.3390/molecules25030536)
Supplement: Supplementary file 1 [file molecules-25-00536-s001.pdf]

molecules

Supporting information

Deep oxidative desulfurization of fuels in presence of Brönsted acidic polyoxometalate-based ionic liquids

Argam Akopyan [1], Ekaterina Eseva [1], Polina Polikarpova [1], Anastasia Kedalo [1], Anna Vutolkina [1,2] and Aleksandr Glotov [1,2,*]

[1] Department of Petroleum Chemistry and Organic Catalysis, Moscow State University, 119991 Moscow, Russia; arvchem@yandex.ru (A.A.); polikarpova-polina@rambler.ru (P.P.); esevakatya@mail.ru (E.E.); nastya.kedalo@mail.ru (A.K.); annavutolkina@mail.ru (A.V.); glotov.a@gubkin.ru (A.G.)
[2] Department of Physical and Colloid Chemistry, Gubkin Russian State University of Oil and Gas, 119991 Moscow, Russia; annavutolkina@mail.ru (A.V.); glotov.a@gubkin.ru (A.G.)
* Correspondence: glotov.a@gubkin.ru

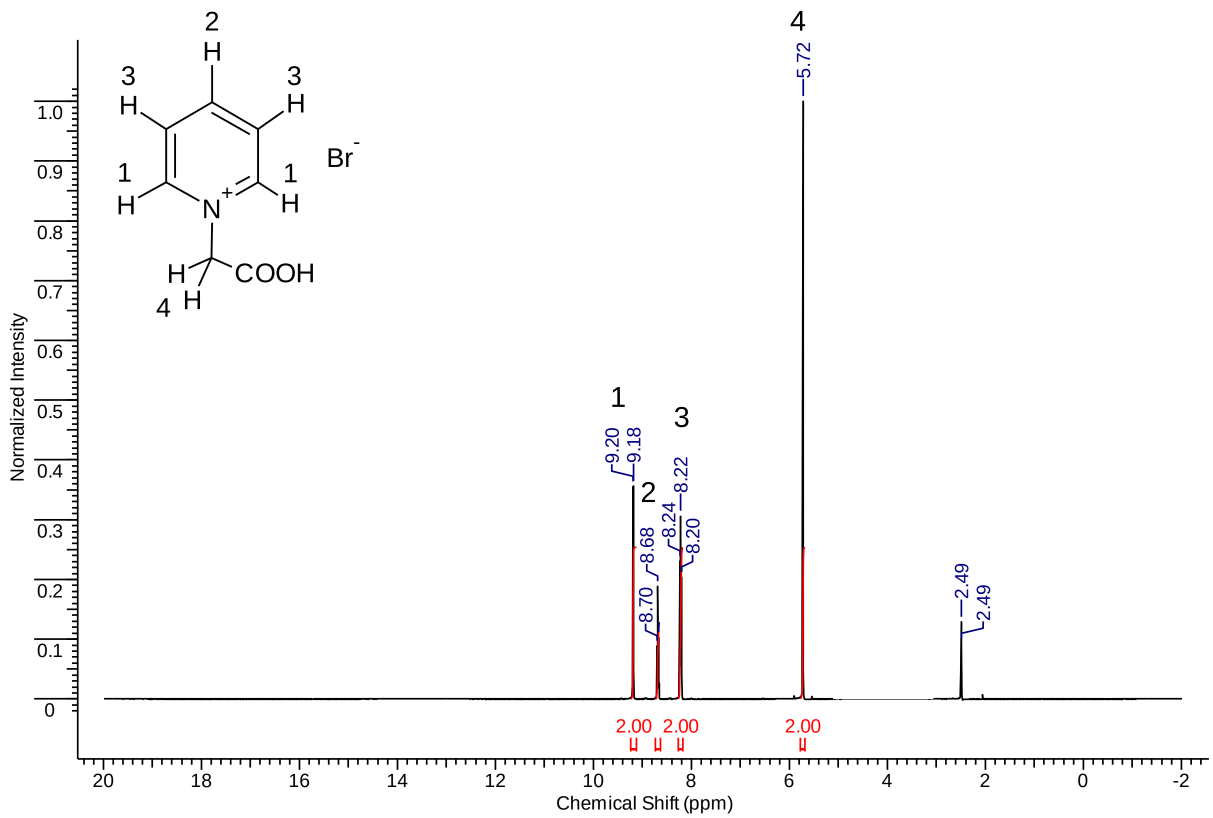

Figure 1. NMR H^1 spectra 1-(carboxymethyl) pyridinium bromide.

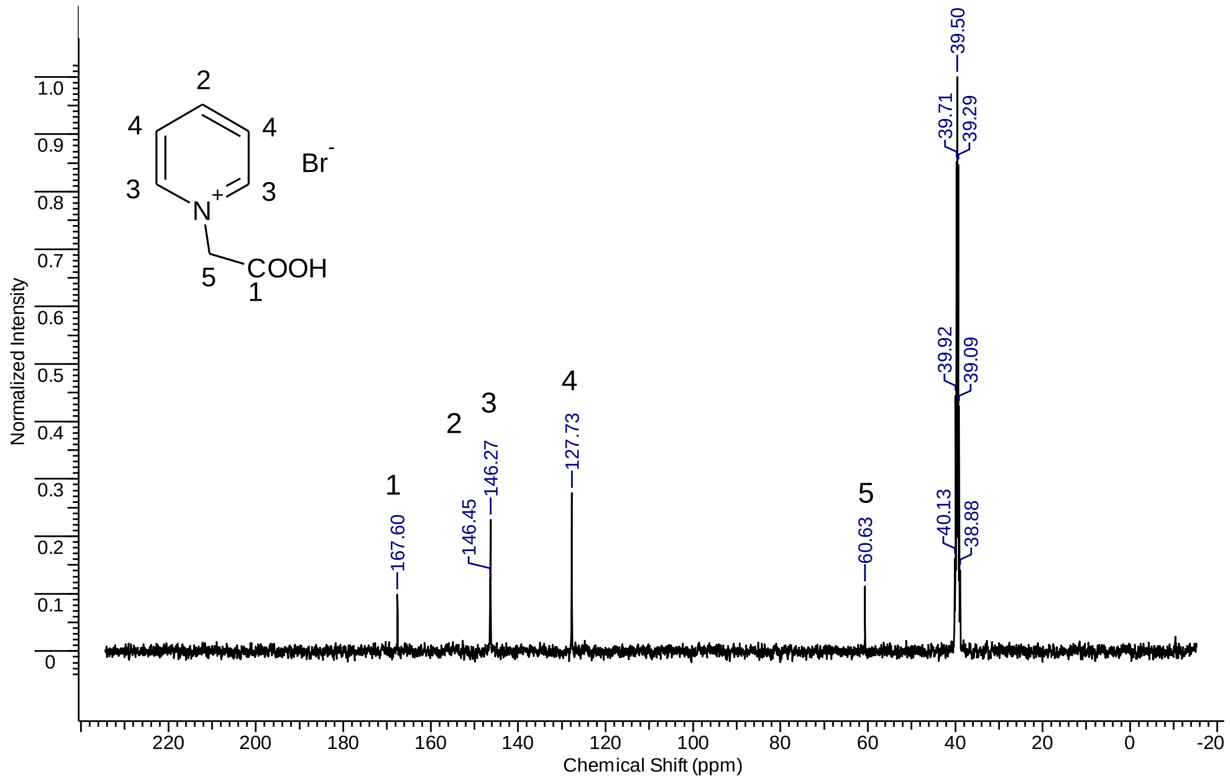

Figure 2. NMR C^{13} spectra 1-(carboxymethyl) pyridinium bromide.

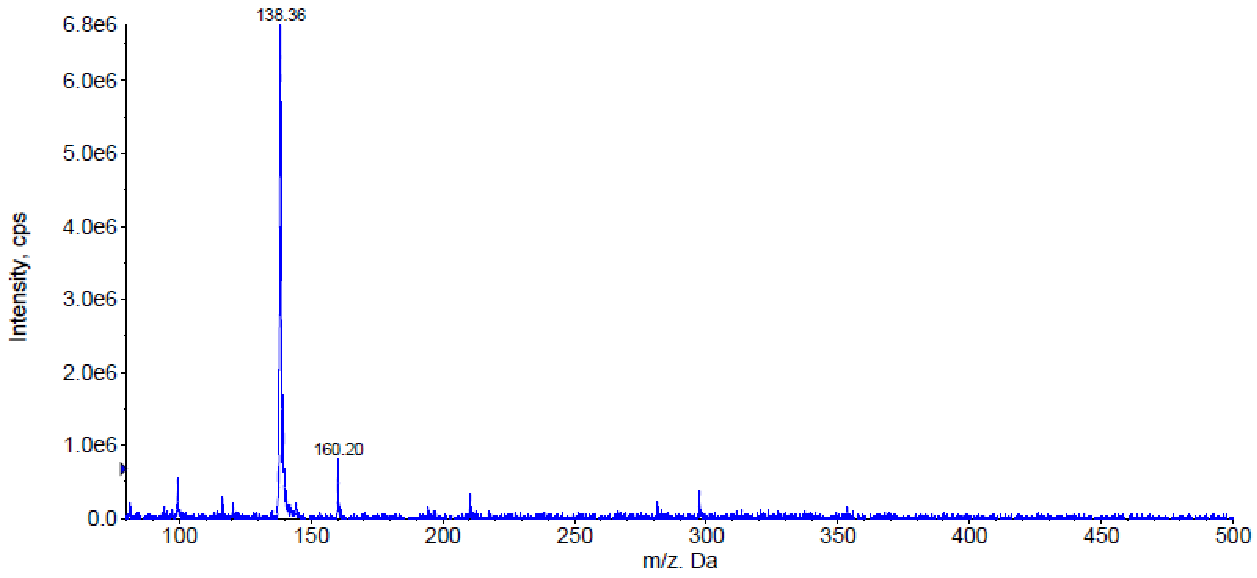

Figure 3. ESI-MS spectra 1-(carboxymethyl) pyridinium bromide.

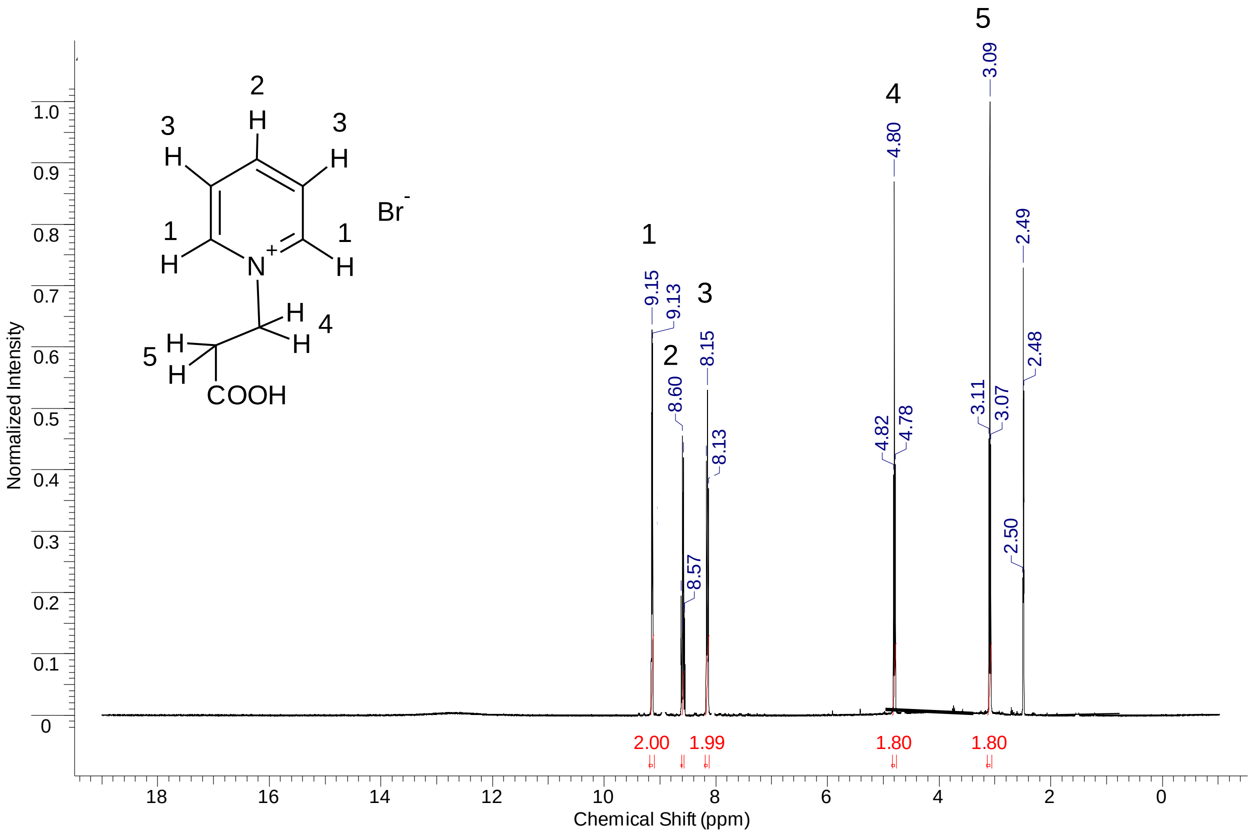

Figure 4. NMR H¹ spectra 1-(2-carboxyethyl) pyridinium bromide.

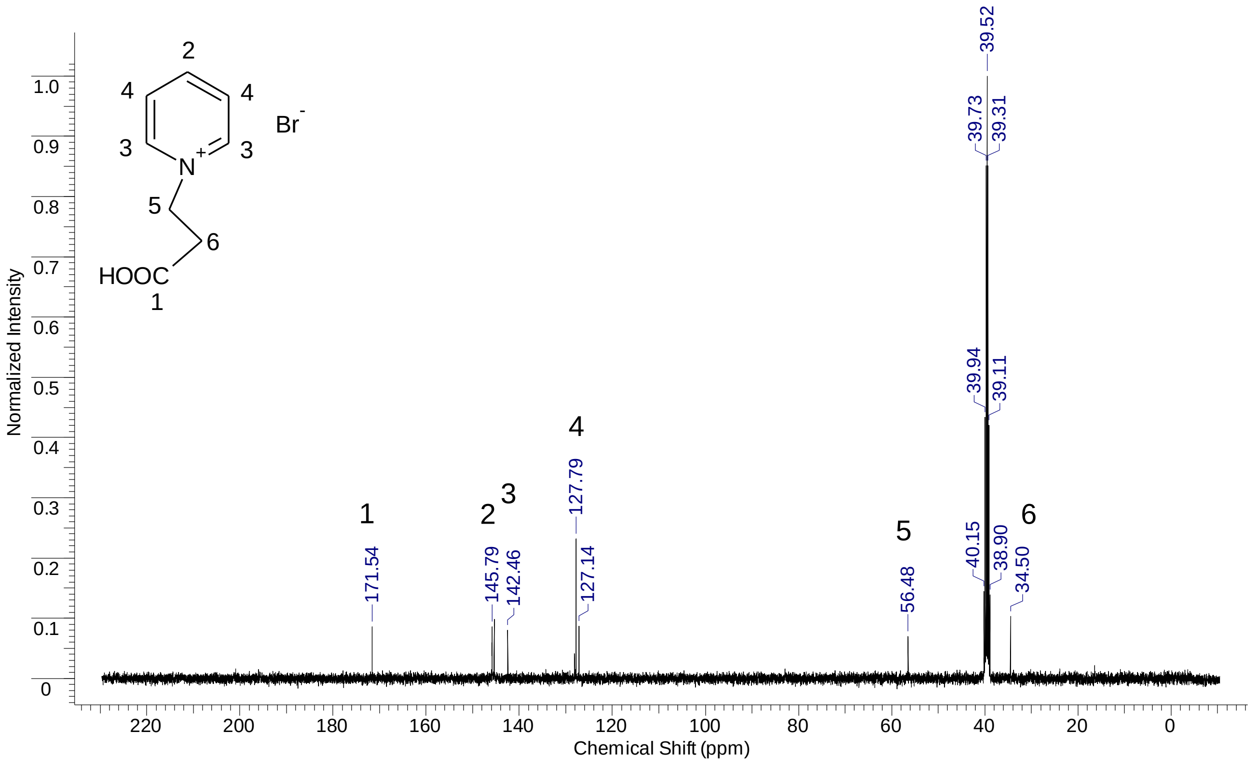

Figure 5. NMR C¹³ spectra 1-(2-carboxyethyl) pyridinium bromide.

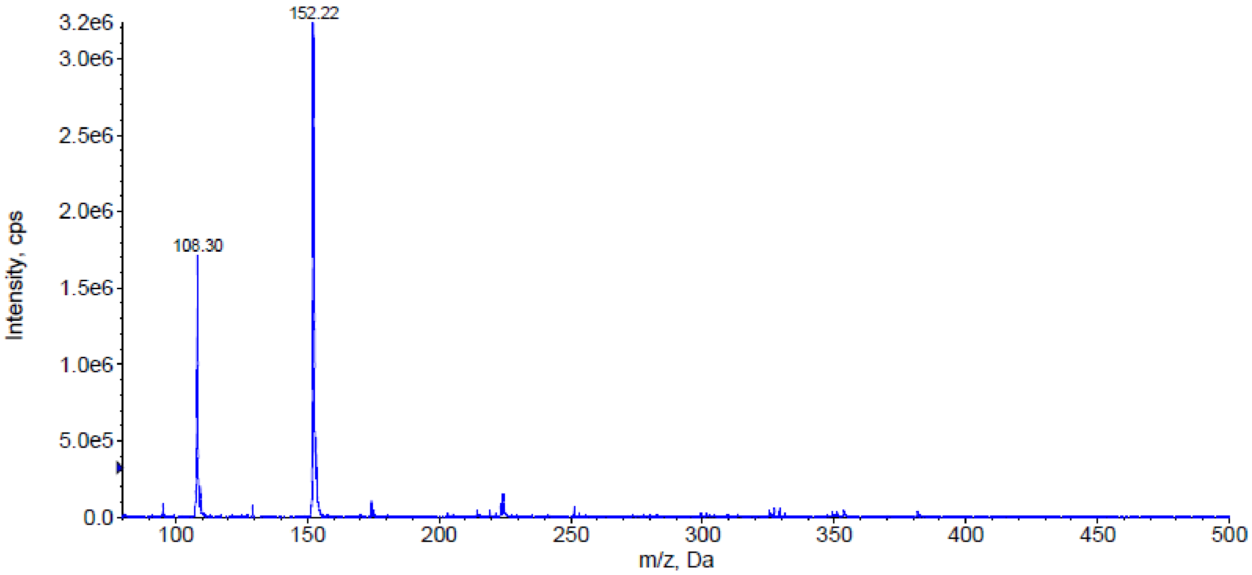

Figure 6. ESI-MS spectra 1-(2-carboxyethyl) pyridinium bromide.

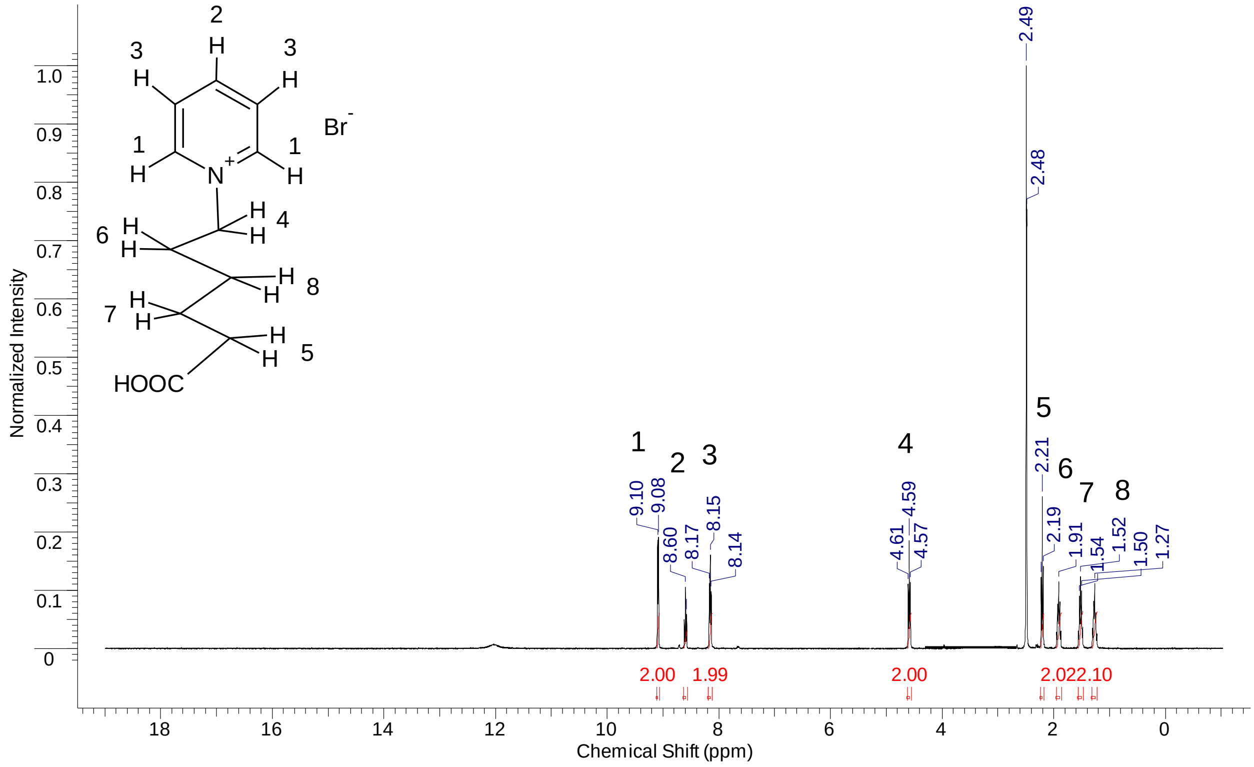

Figure 7. NMR H¹ spectra 1-(5-carboxypentyl) pyridinium bromide.

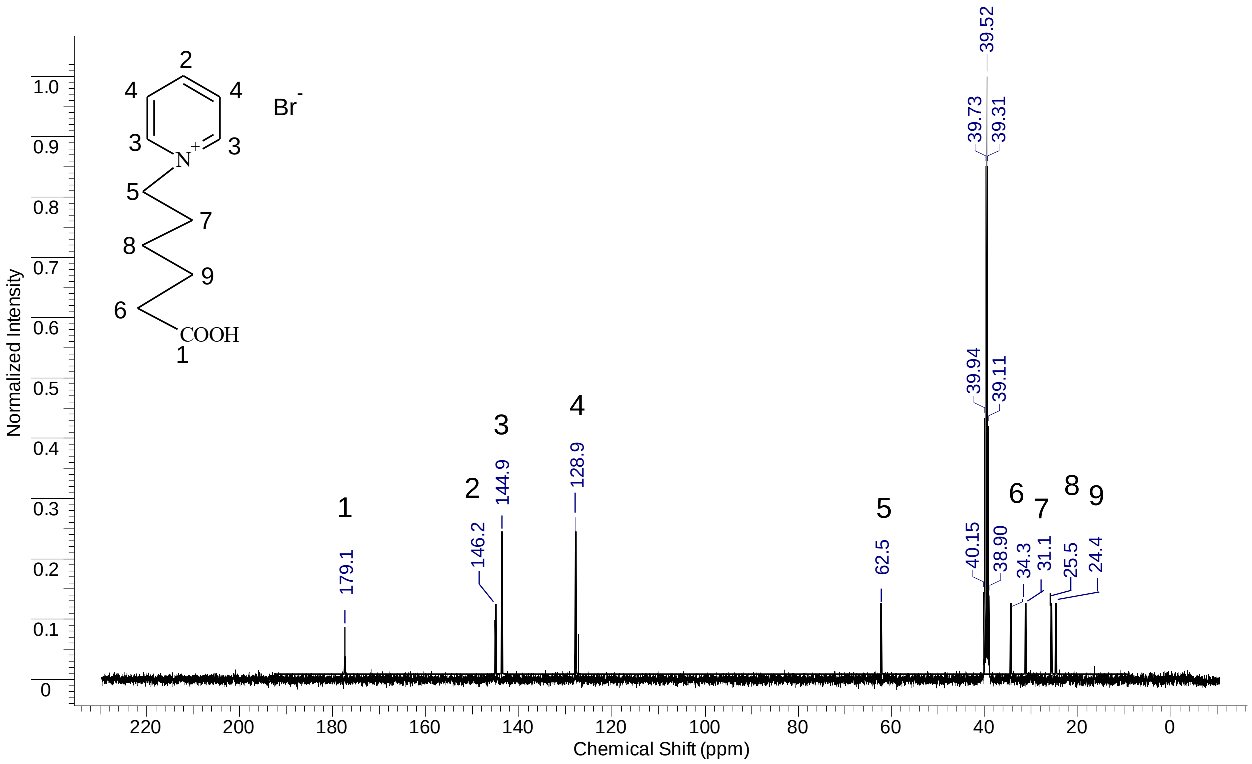

Figure 8. NMR C^{13} spectra 1-(5-carboxypentyl) pyridinium bromide.

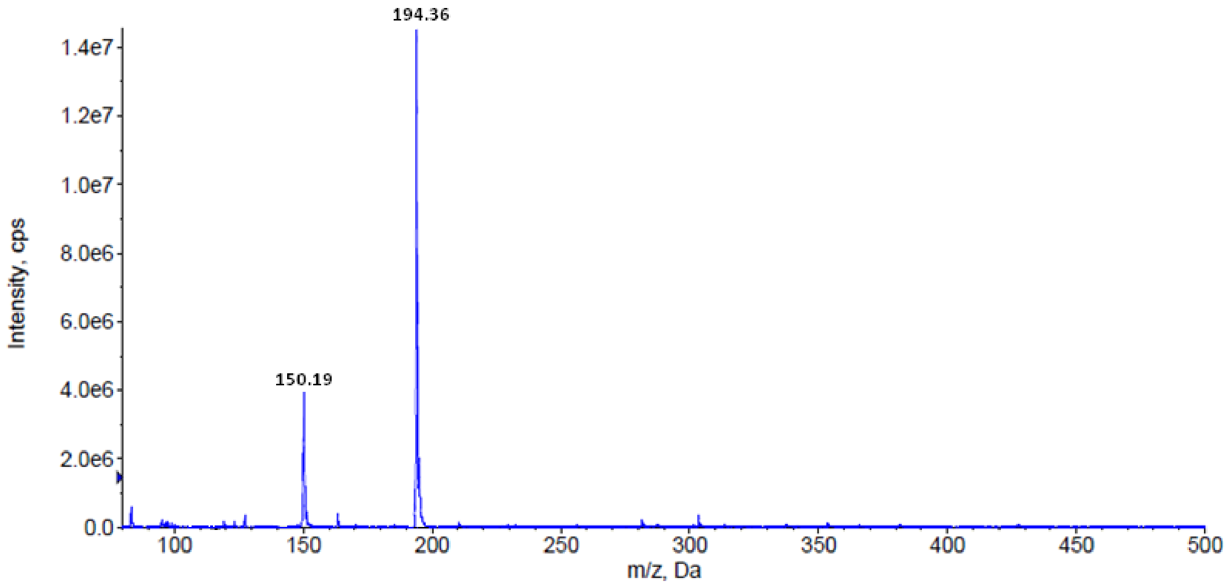

Figure 9. ESI-MS spectra 1-(5-carboxypentyl) pyridinium bromide.

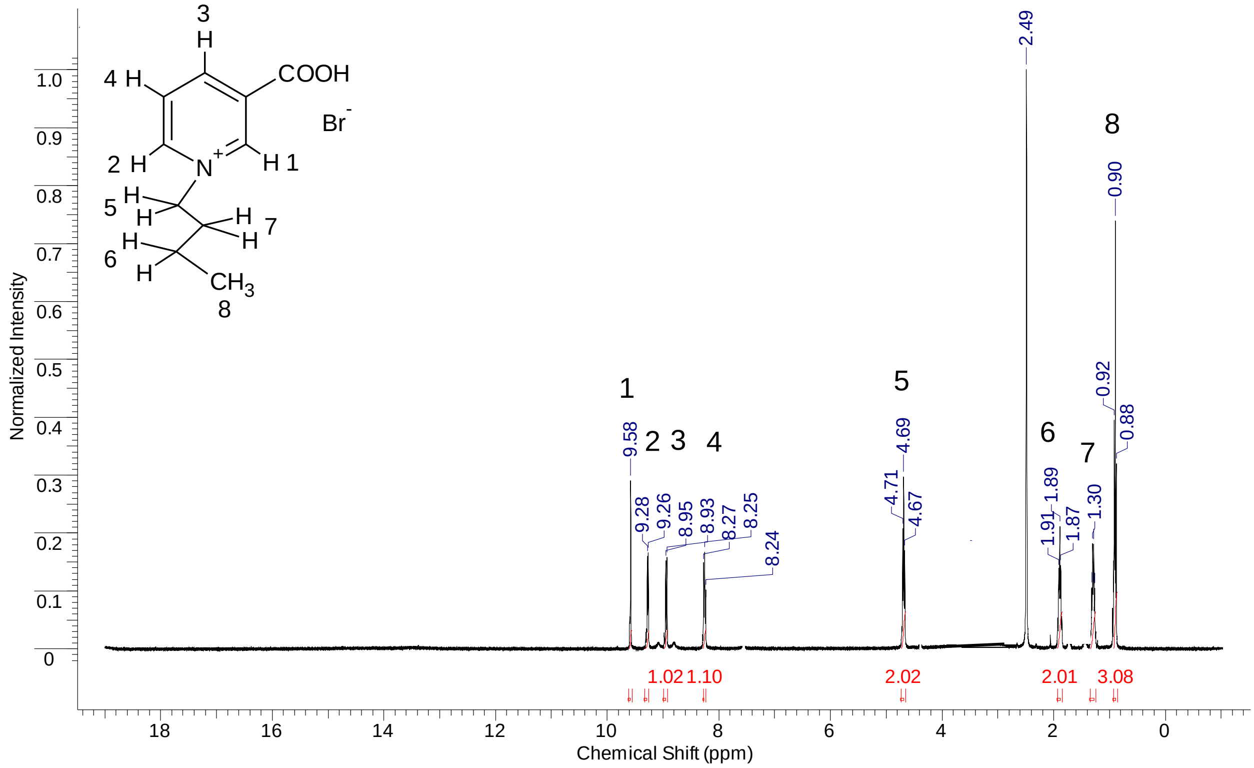

Figure 10. NMR H¹ spectra 1-butyl-3-carboxypyridinium bromide.

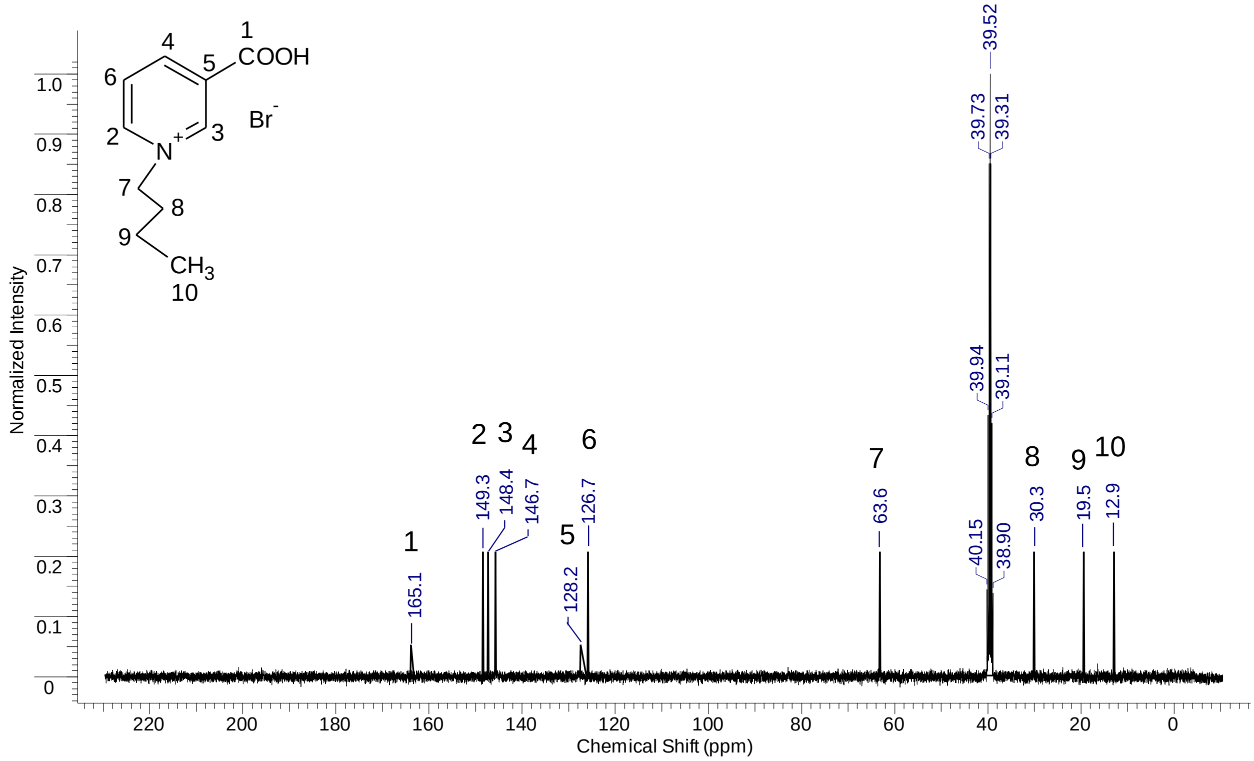

Figure 11. NMR C¹³ spectra 1-butyl-3-carboxypyridinium bromide.

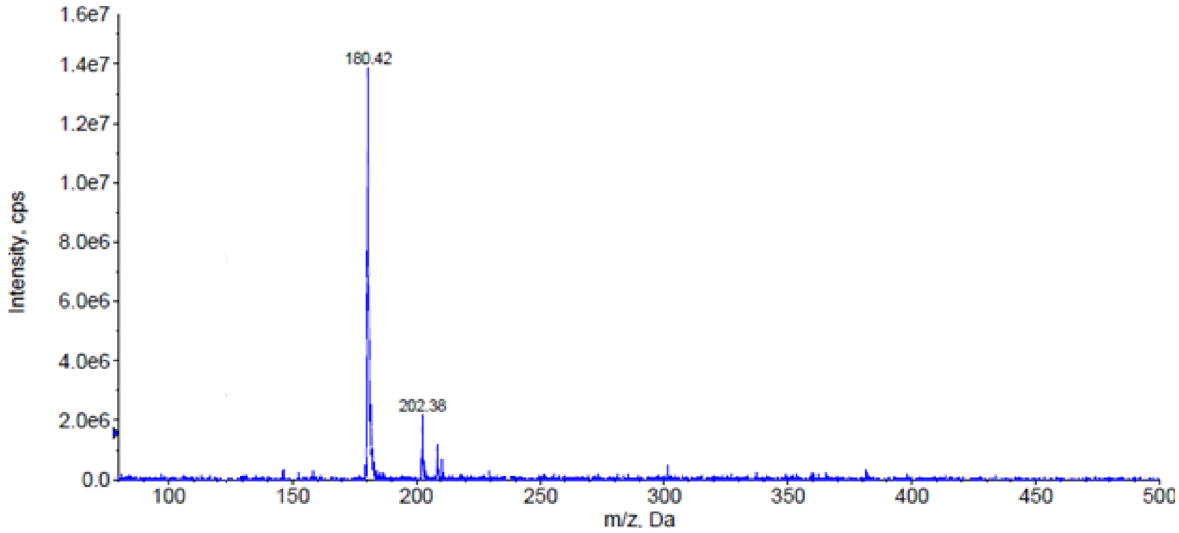

Figure 12. ESI-MS spectra 1-butyl-3-carboxypyridinium bromide.

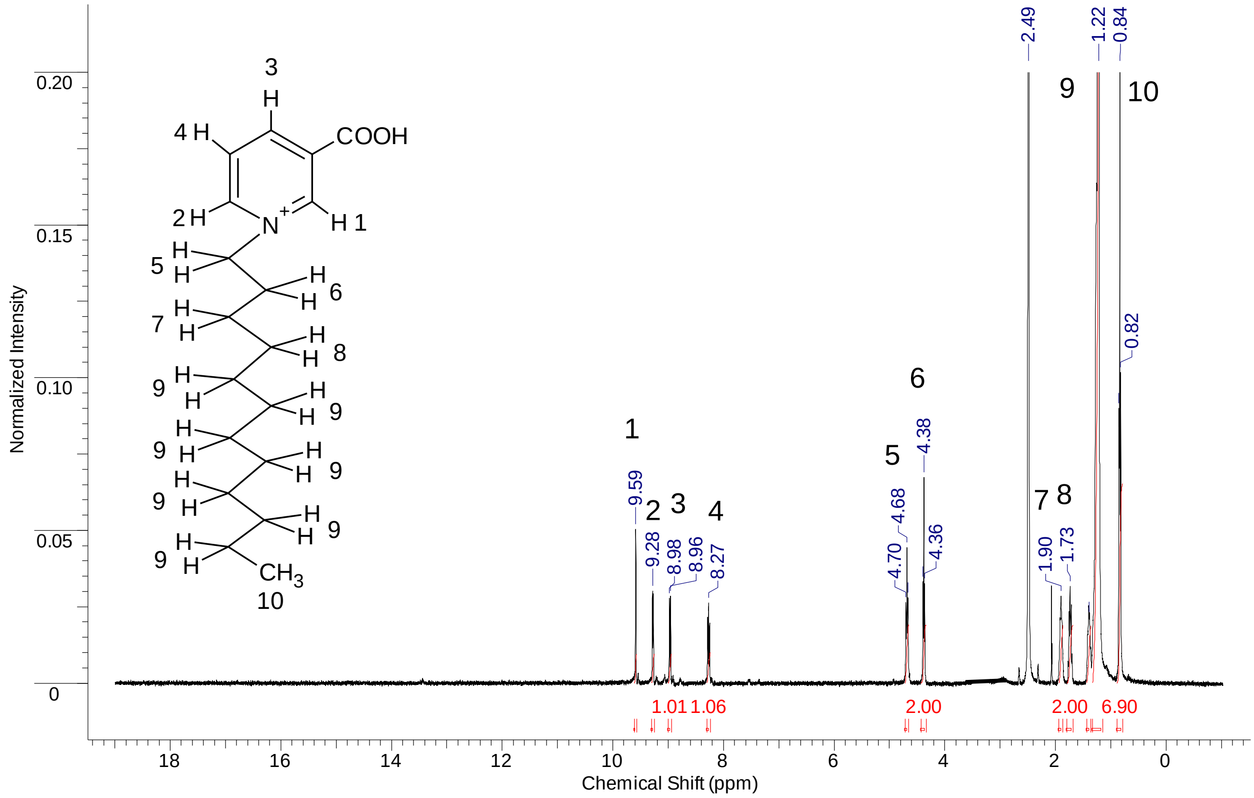

Figure 13. NMR H¹ spectra 1-dodecyl-3-carboxypyridinium bromide

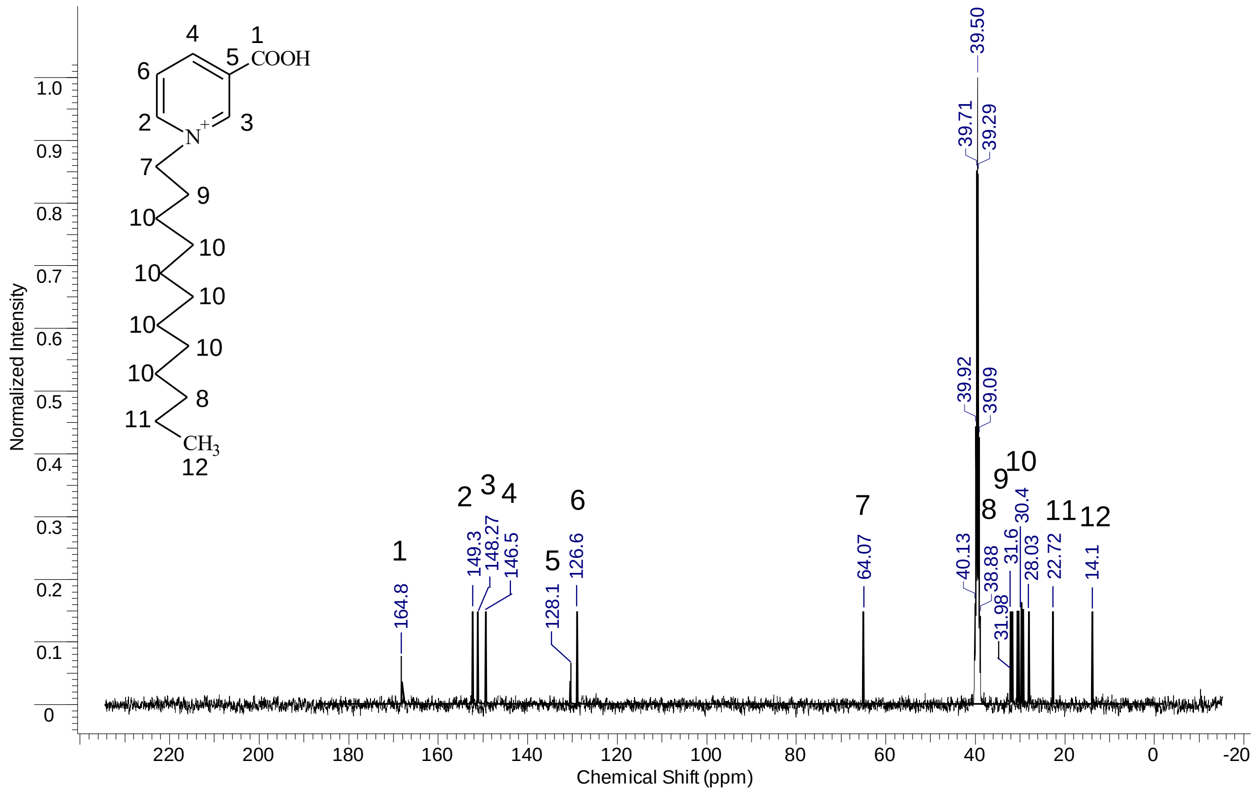

Figure 14. NMR C[13] spectra 1-dodecyl-3-carboxypyridinium bromide.

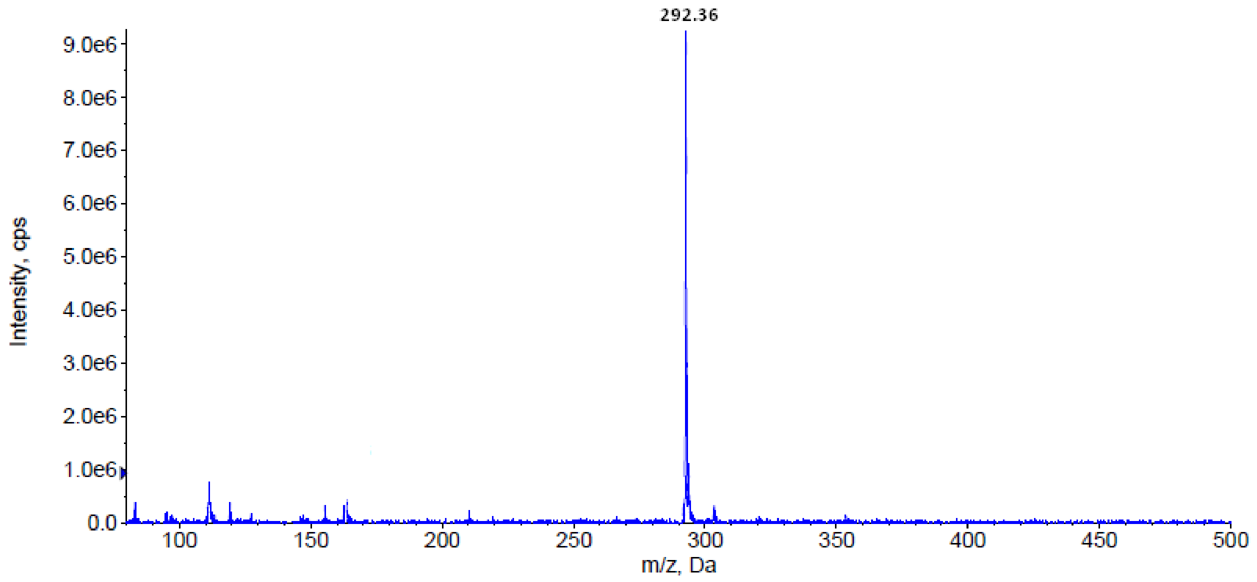

Figure 15. ESI-MS spectra 1-dodecyl-3-carboxypyridinium bromide.

© 2020 by the authors. Submitted for possible open access publication under the terms and conditions of the Creative Commons Attribution (CC BY) license (http://creativecommons.org/licenses/by/4.0/).
